# The Connection Between Paid and Unpaid Supports With Person-Centered Outcomes for Older Adults With Disabilities

**DOI:** 10.1093/geroni/igaf122.1310

**Published:** 2025-12-31

**Authors:** Lindsay Dubois, Rosa Plasencia, Stephanie Giordano, Nilufer Isvan

**Affiliations:** Human Services Research Institute, Cambridge, Massachusetts, United States; ADvancing States, Arlington, Virginia, United States; Human Services Research Institute, Cambridge, Massachusetts, United States; Human Services Research Institute, Cambridge, Massachusetts, United States

## Abstract

As funding for long-term services and supports shifts from institutional settings to home and community-based services (HCBS), the need for consumer-reported outcomes data is paramount. This study uses data from National Core Indicators—Aging and Disabilities™ (NCI-AD™) Adult Consumer Survey (ACS) to investigate the connection between types of providers (e.g., paid staff, paid family, unpaid supports) on self-reported service experiences of adults receiving HCBS, controlling for relevant service and recipient characteristics. The unadjusted likelihood of having sufficient help with activities of daily living (ADL) is highest among people whose primary caregiver is a paid family member compared to paid nonfamily staff (OR = 2.26, p < 0.001) and lowest for unpaid family caregivers (OR = 0.71, p < 0.001). Likewise, the unadjusted likelihood of reporting that services and supports meet needs was highest among those whose primary caregiver is a paid family member compared to paid nonfamily staff (OR = 1.42, p < 0.001) and lowest for unpaid family caregivers (OR = 0.77, p < 0.001). Adjusting for covariates, the likelihood of having sufficient help with ADL and services meeting needs remains significantly higher for those with paid family caregiver relative to paid staff (aOR=1.66, p < 0.001, and aOR=1.42, p = 0.002 respectively). These data suggest that even with the influence of demographic and service-related factors, paid family caregivers are associated with positive outcomes for older adults and people with disabilities using HCBS. These results can support policy and practice that workforce needs, improving benefits to reduce turnover, and implementing caregiver support initiatives.

